# Supplementary figures and images for: MicroRNA-206 suppresses growth and metastasis of breast cancer stem cells via blocking EVI-1-mediated CALR expression
Source: PLoS One. 2022 Sep 22;17(9):e0274919. doi: 10.1371/journal.pone.0274919 (PMC9498949; doi:10.1371/journal.pone.0274919)

Fig.1

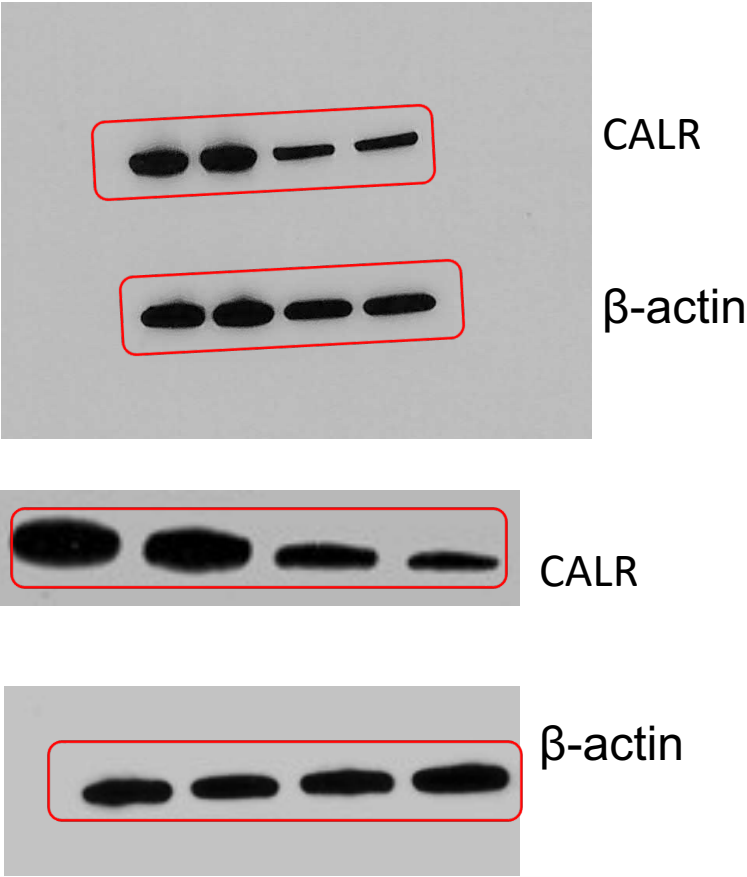

Fig.2

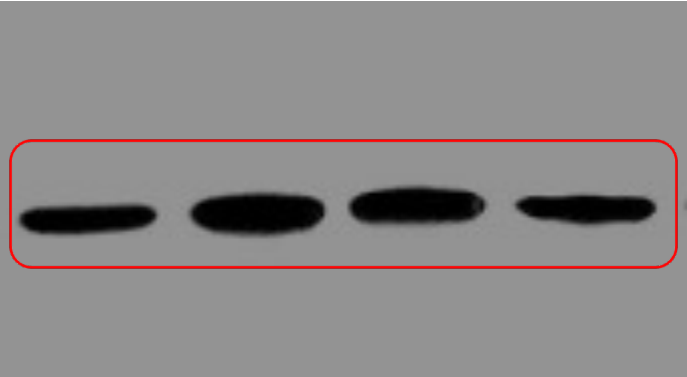

CALR

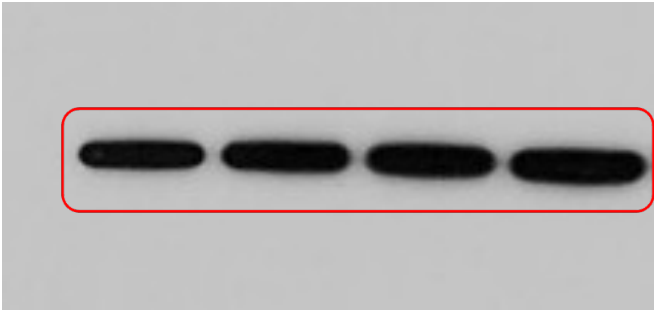

$\beta$ -actin

Fig.3

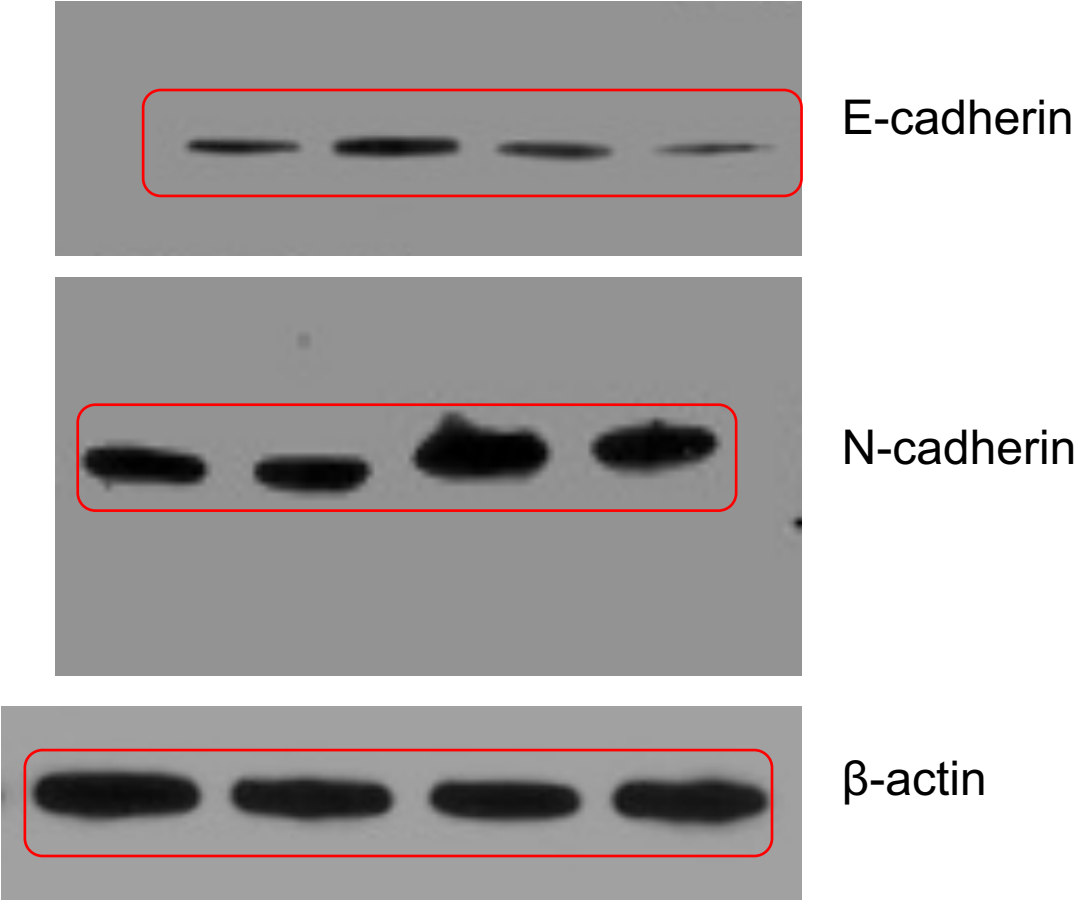

Fig.4

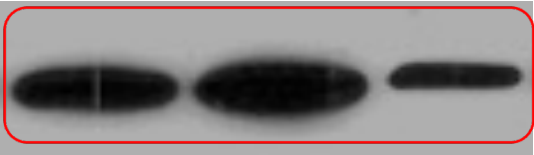

CALR

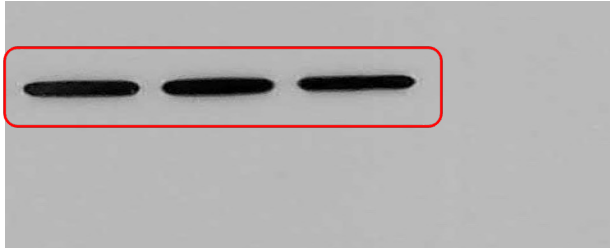

β-actin

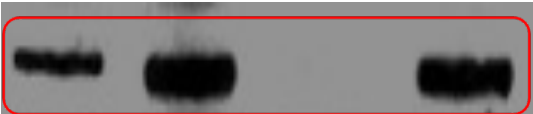

EVI-1

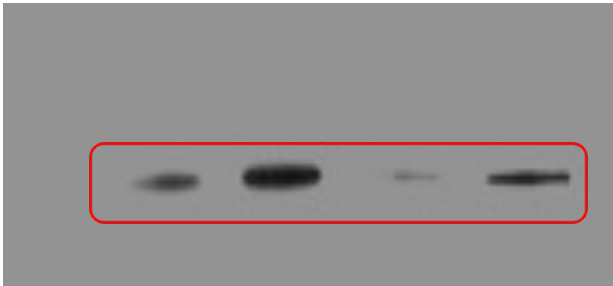

CALR

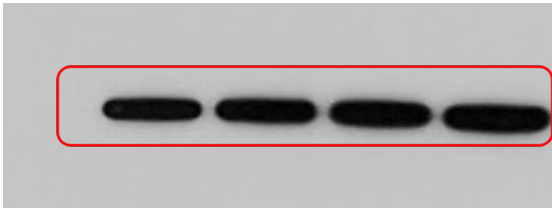

β-actin

Supplement: S1 File — (PDF) [file pone.0274919.s001.pdf]
